# Obstetric outcomes of young women following in-vitro fertilization: a case–control study

**DOI:** 10.1186/s12884-022-04502-8

**Published:** 2022-02-28

**Authors:** Gilad Karavani, Henry H. Chill, Aharon Dick, Marva Bergman, Tal Imbar, Sorina Grisaru-Granovsky, Assaf Ben-Meir

**Affiliations:** 1 Infertility and IVF Unit, Hadassah Ein-Kerem Medical Center and Faculty of Medicine, Hebrew University of Jerusalem, Jerusalem, Israel; 2 Department of Obstetrics and Gynecology, Hadassah Ein-Kerem Medical Center and Faculty of Medicine, Hebrew University of Jerusalem, Jerusalem, Israel; 3 Division of Urogynecology, University of Chicago Pritzker School of Medicine, NorthShore University HealthSystem, Skokie, IL USA; 4 Department of Obstetrics and Gynecology, Shaare Zedek Medical Center and Faculty of Medicine, Hebrew University of Jerusalem, Jerusalem, Israel

**Keywords:** Assisted reproductive technology, In-vitro fertilization, Perinatal outcome, Young women

## Abstract

**Background:**

Women undergoing in-vitro fertilization (IVF) treatments are at increased risk for maternal and neonatal complications compared to women who conceive spontaneously. Though spontaneous pregnancies of young women and adolescents have an increased risk for adverse maternal and neonatal outcomes, pregnancy outcomes of this age group, following IVF treatment have been scarcely reported. The aim of this study was to report maternal and neonatal outcomes of young women who conceived following IVF compared to women in the same age group with spontaneous conception.

**Methods:**

We performed a multicenter case–control study. The study group included women aged 17–25 years who conceived by IVF with an ongoing singleton pregnancy. For the purpose of the study, a control group matched (1:2 ratio) for maternal age at delivery and parity was constructed. Demographic, medical history, pregnancy related characteristics and maternal and neonatal outcomes were compared between groups. Finally, factors associated with spontaneous vaginal delivery were assessed for the entire cohort using a univariate and multivariate logistic regression model.

**Results:**

Between 2005 and 2021, we identified 80 women aged 19–25 years who conceived by IVF. A control group of 160 women was matched to the study group by age and parity. The unmatched maternal characteristics and pregnancy associated complications were similar among the groups. However, the IVF group had a significantly higher rate of induction of labor (48.1% vs. 26.6%, *p* = 0.001), meconium-stained amniotic fluid (27.6% vs. 14.1%, *p* = 0.025), prolonged second stage of labor (26.0% vs. 7.3%, *p* = 0.001) and operative vaginal delivery (22.5% vs.12.5%, *p* = 0.048).

Neonatal outcomes were for the most part comparable; nevertheless, we found a higher rate of neonates with an umbilical artery pH < 7.1 in the IVF group (9.8% vs. 0.0%, respectively; *p* = 0.022).

A logistic regression analysis for spontaneous vaginal delivery (vs. cesarean or operative vaginal deliveries) found that spontaneous onset of labor (vs. induction of labor) (OR = 2.08; 95% CI = 1.07–4.05, *p* = 0.03) was positively associated with spontaneous vaginal delivery while prolonged second stage of labor (OR = 0.35; 95% CI = 0.13–0.95, *p* = 0.04) was negatively associated with this parameter.

**Conclusion:**

Young women who conceive by in-vitro fertilization are expected to reach favorable pregnancy outcomes, comparable to women who conceived spontaneously.

## Introduction

The use of Assisted reproductive technology (ART) is on the rise worldwide. It is estimated that two percent of all births in the United States follow treatment using ART, and this number is estimated to increase in coming years [1, 2].

Numerous studies have shown women undergoing ART and specifically in-vitro fertilization (IVF), are at increased risk for maternal and neonatal complications compared to their counterparts [3–7]. Hypertensive disorders of pregnancy, gestational diabetes, low birth weight, preterm delivery and placental complications have all been associated with subfertility and IVF treatment [8–10]. To date it is still unclear whether underlying infertility or adverse effects brought upon by the infertility treatment are the main cause for these findings.

Since most patients undergoing IVF are above 35 years of age, young women with infertility are a unique group which has not been sufficiently studied. The few studies which have focused on this age group have found reproductive outcomes to be less favorable compared to other age groups [11, 12]. Furthermore, they have reported mainly on fertility outcomes rather than perinatal ones.

The aim of this study was to report on obstetric and neonatal outcomes in young women undergoing IVF treatment.

## Materials and methods

### Study population and data collection

This was a case–control study conducted at three University-affiliated hospitals: Hadassah Medical Center, which includes two campuses and Shaare Zedek Medical Center between August 2005 and July 2021. The study group included women aged 17–25 years who underwent controlled ovarian hyperstimulation (COHS) and embryo transfer (at Hadassah Medical Center only), which resulted in a clinical pregnancy (defined by an ultrasound measurement at 6 weeks of pregnancy or later with evidence of fetal heart activity). Pregnancies were followed-up in the fertility unit until 6–8 weeks of gestation and then transferred to an outpatient clinic. Labor related data was collected from electronic medical records of the three medical centers – two Hadassah Medical Center campuses and Shaare Zedek Medical Center. For the purpose of the study, we excluded cases of miscarriage, ectopic pregnancy, deliveries after maternal age of 26 years, multifetal pregnancy or patientsfor whom obstetric data was unavailable.

A control group was identified including women who conceived spontaneously with a single fetus. Matching was performed for age, parity and medical center in which the delivery took place at a ratio of 1:2.Similar exclusion criteria were implemented for the control group.

General medical history, pregnancy and labor and delivery characteristics and outcomes were collected. Maternal and neonatal data were retrieved using a computerized database, continuously updated and validated for admission, labor, and postpartum course.

The study protocol was approved by the Institutional Review Boards:

Hadassah Medical Center (IRB number 0714–20-HMO) and shaare Zedek Medical Center (IRB number 0158–21-SZMC).

### Reproductive, IVF protocol and embryo transfer data

Reproductive data of the study group was collected from the electronic medical records of the IVF unit. This data included age at cycle initiation, age at menarche, infertility etiology, duration of infertility, gravidity, parity, previous miscarriages and number of previous IVF cycles and IVF cycle’s outcomes.

The COHS protocol, ovum pick-up, fertilization technique and embryo culture and transfer have been previously described [13]. Briefly, COHS was initiated by gonadotropin-releasing hormone (GnRH) long or short protocols or GnRH antagonist protocol. The long GnRH agonist protocol included administration of 0.1 mg GnRH agonist on cycle-day 21, followed by daily administration of gonadotropins starting on cycle-day 2–3, while the short GnRH agonist protocol included administration of 0.1 mg GnRH agonist on cycle-day 1, followed by daily administration of gonadotropins starting on cycle-day 2–3. The GnRH antagonist protocol included daily administration of gonadotropins starting on cycle-day 2–3 followed by a subcutaneous administration of GnRH antagonist when follicles size reached 14 mm (mm) or on the 6th day of gonadotropin treatment. In all three protocols ovulation induction was achieved by hCG or GnRH agonist injections given when follicles reach 16 to 18 mm in size. Ovum pick up was performed under general anesthesia using trans-vaginal ultrasound guidance 34–36 h after ovulation induction.

Embryo quality was determined by cell number, symmetry and fragmentation and was graded “A”,” B” or “C” according to the SART grading. Embryos were cultured in a one-step medium ("SAGE 1-step" (SAGE, Al-rad medical, Nes Ziona, Israel). Fresh embryo transfer was performed on day 2, day 3, or at blastocyst stage according to patient and laboratory convenience as well as embryo quality and number of good quality embryos. Embryo transfer was performed with abdominal ultrasound (US) guidance using one of two catheters—the SIVF catheter (K-Jets-7019-SIVF; Cook IVF, Eight Miles Plains, Queensland, Australia) or the Edwards-Wallace catheter (Classic Embryo Replacement Catheter; Smiths Medical, Hythe, Kent, U.K.) upon operator preference.

Frozen thawed embryo transfers were performed in artificial cycles using exogenous estradiol and vaginal progesterone or in natural cycles. In the artificial cycles, patients started 17β-E2 6 mg/day given orally in 3 divided doses on the first day of their menstrual cycle for a week, with a similar dose of Estrofem for additional 5–13 days given after US and blood tests. Once endometrial thickness measurment reached 8 mm, micronized progestrone pills were added for 2–5 days prior to embryo transfer.

### Pregnancy, obstetric and neonatal characteristics and outcomes

Maternal general and obstetric data included age, gravidity, parity, previous miscarriages and ectopic pregnancies, previous vaginal and cesarean deliveries, pre-gestational and gestational diabetes mellitus, hypertensive disorders of pregnancy and gestational age at delivery (weeks).

Intra-partum characteristics of the study population included data regarding mode of labor initiation (spontaneous or induction of labor), artificial rupture of membranes, analgesia/anesthesia type, duration of 1^st^ and 2^nd^ stages of labor, rate of prolonged 2^nd^ stage of labor (over 2 h without epidural analgesia or 3 h with epidural analgesia in nulliparous women and 1 h less in parous women) [14], intra-partum fever (above 38 °C in two consecutive measurements) [15], meconium stained amniotic fluid, mode of delivery (spontaneous vaginal delivery, operative vaginal delivery, elective and urgent cesarean section), rate of post-partum hemorrhage (defined as more than 500 ml at vaginal delivery or more than 1000 ml during cesarean delivery), shoulder dystocia and episiotomy.

Neonatal data included birthweight (grams), low birthweight < 2500 g (LBW), gender, Apgar score at 1st and 5th minute, admission to the neonatal intensive-care unit (NICU), pH level at delivery (when available) and perinatal mortality (including antepartum, intrapartum and postpartum death).

### Statistical analysis

Testing the association between two categorical variables was carried out using either the Chi-Square test or the Fisher’s exact test, as indicated. The Fisher’s exact test was applied in analyses of small samples, when more than 20% of cells have expected frequencies of less than 5. Quantitative variables were compared using the student’s t-test for the two independent groups or the Mann–Whitney U-test. The student’s t-test was used for normally distributed parameters, while the Mann–Whitney U-test was done for non-normally distributed parameters samples.

Univariate analysis was performed to identify factors associated with spontaneous vaginal delivery. Descriptive univariate analyses were performed accordingly. Variables found significantly associated with the dependent variable of spontaneous vaginal delivery in the univariate analysis, were entered into the multivariate logistic regression model. The significance of each variable and the adjusted Odds Ratio and 95% confidence interval (OR, 95%CI) were calculated. A *p*-value of < 0.05 was considered statistically significant for all comparisons.

## Results

**Fig. 1:**
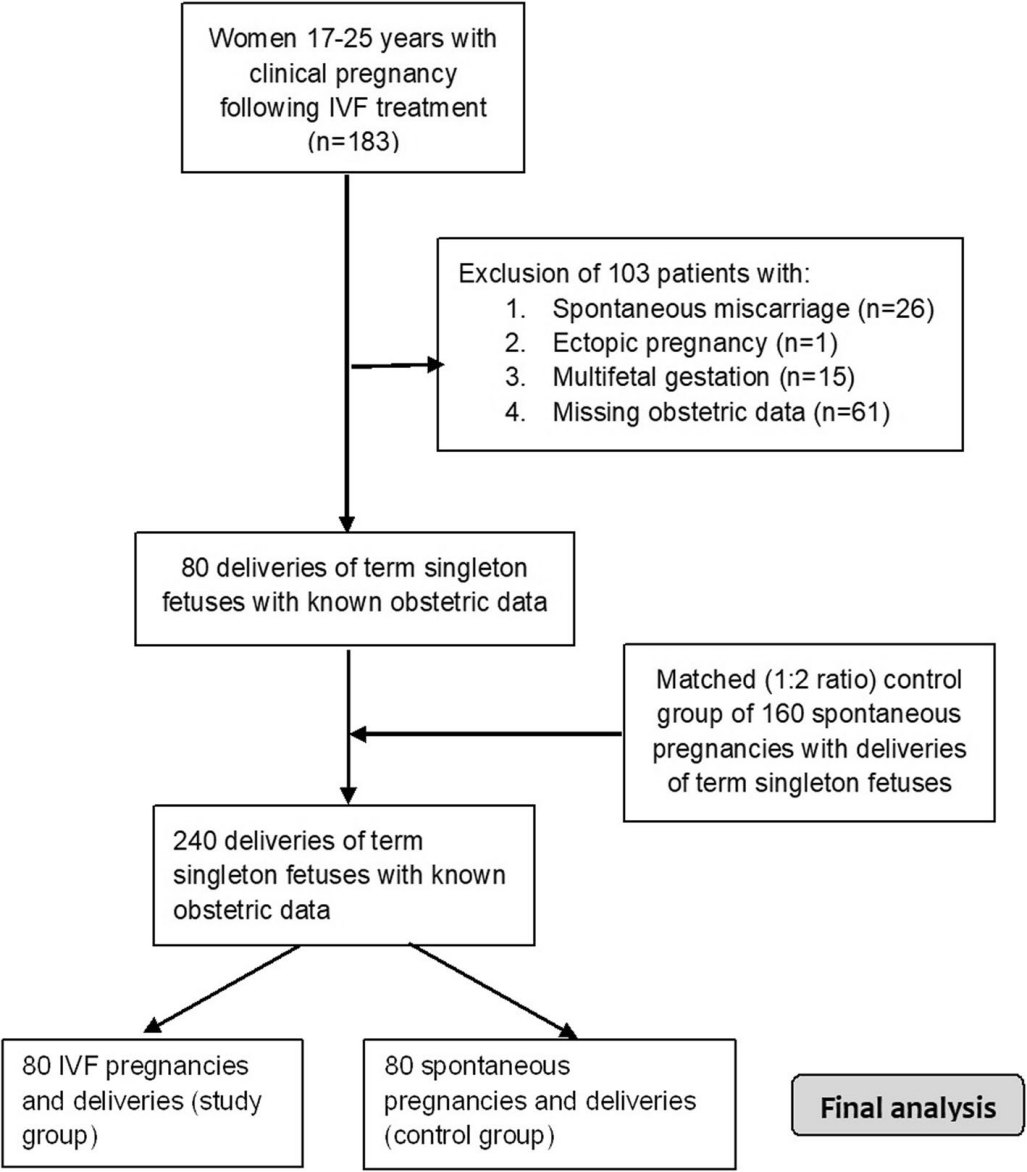
Flow chart of the study population

During the study period, 183 women aged 17–25 years were identified who underwent IVF treatment and achieved clinical pregnancy. Twenty-six cases (14.2%) which ended with a spontaneous miscarriage, one ectopic pregnancy and fifteen women (8.2%) with multifetal pregnancy (14 twin pregnancies and one triplet pregnancy) were excluded. Pregnancy and obstetric related information for 80 women (80/141 (56.7%)) in the IVF group were obtained (Fig. 1). The maternal age ranged between 19–25 years.

Male factor infertility was the most common diagnosis (63.8%), followed by pre-implantation genetic testing (12.5%), combined female and male etiology (8.8%), ovulation disorder (6.3%), unexplained infertility (6.3%) and endometriosis (2.5%).

Mean age for the entire cohort (IVF and spontaneous pregnancy groups) was 23.7 ± 1.3 years. Nulliparity rate was 85% and the median gestational age was 39 ^0/7^ weeks with a mean birthweight of 3065.3 ± 532.4 g. The matched control spontaneous pregnancy group (*n* = 160) did not differ in maternal characteristics including previous miscarriages and previous vaginal and cesarean deliveries (Table 1). Moreover, the IVF group did not demonstrate higher rates of early or late pre-term deliveries and had similar rates of gestational diabetes and pregnancy induced hypertension.

**Table 1:** Demographic and obstetric characteristics of the study population- the study (IVF pregnancy) and control (spontaneous pregnancy) groups

Parameter	IVF pregnancy (*n*=80)	Spontaneous pregnancy (*n*=160)	*P* value
**Age**	23.6±1.3	23.7±1.3	0.408
**Gravidity **			0.24
**1**	62 (77.5%)	134 (83.8%)	
**2 or more**	18 (22.5%)	26 (16.3%)	
**Parity**			1
**0**	74 (92.5%)	148 (92.5%)	
**1**	4 (5.0%)	8 (5.0%)	
**2 or more**	2 (2.5%)	4 (2.5%)	
**Previous miscarriages**			0.051
**0**	69 (86.3%)	149/156 (95.5%)	
**1**	7 (8.8%)	6/156 (3.9%)	
**2**	3 (3.8%)	1/156 (0.6%)	
**3 or more**	1 (1.3%)	0	
**Previous vaginal delivery**	6 (92.5%)	11 (6.9%)	0.572
**Previous cesarean delivery**	0	1 (0.6%)	0.667
**Gestational diabetes**	0	4 (2.5%)	0.195
**PIH/Preeclampsia**	2 (2.5%)	2 (1.3%)	0.407
**Gestational week**	38.7±2.5 (39.2 (37.8-40.1))	39.0±1.8 (39.0 (38.0-40.0))	0.354
**Term delivery (>37 weeks)**	69 (86.3%)	148 (9.3%)	0.121
**Late preterm (34-37 weeks)**	8 (10.0%)	9 (5.6%)	0.285
**Early preterm (<34 weeks)**	3 (3.4%)	3 (1.9%)	0.403

Data presented as mean ± SD, mean ± SD (median (Q1-Q3)) (for non-normally distributed parameters) or n(%)

*Note: IVF* In-Vitro Fertilization, *PIH* Pregnancy Induced Hypertension

A comparison of maternal obstetric outcomes between the groups showed several significant differences (Table 2). Women in the IVF group had an increased rate of induction of labor (48.1% vs. 26.6%, *p* = 0.001), occurrence of meconium-stained amniotic fluid (27.6% vs. 14.1%; *p* = 0.025), longer overall duration of the second stage of labor (1.7 vs. 1.3 h, *p* = 0.034) and higher rate of prolonged second stage of labor (26.0% vs. 7.3%, *p* = 0.001).

**Table 2:** Labor related characteristics of the study (IVF pregnancy) and control (spontaneous pregnancy) groups

**Parameter**	**IVF pregnancy (*n*=80)**	**Spontaneous pregnancy (*n*=160)**	***P* value**
**Induction of labor**	37/77 (48.1%)	42/158 (26.6%)	0.001
**Epidural analgesia**	53/75 (70.7%)	104/160 (65.0%)	0.39
**Artificial rupture of membranes**	32/73 (43.8%)	72/142 (50.7%)	0.34
**Meconium stained amniotic fluid**	16/58 (27.6%)	21/149 (14.1%)	0.025
**Intrapartum fever **^**a**^	4 (5.0%)	9 (5.6%)	0.84
**Duration of 1**^**st**^** stage (hours)**	9.3±6.0	10.3±7.1	0.589
**Duration of 2**^**nd**^** stage (hours)**	1.7±1.3 (1.2 (0.5-3.0))	1.3±1.0 (1.1 (0.5-2.0))	0.034
**Prolonged 2**^**nd**^** stage**	13/50 (26.0%)	10/138 (7.3%)	0.001
**Mode of delivery **			
**Vaginal delivery**	47 (58.8%)	116 (72.5%)	0.033
**Operative vaginal delivery**	18 (22.5%)	20 (12.5%)	0.048
** Cesarean delivery**	15 (18.8%)	24 (15.0%)	0.459
** Elective CD**	4/15 (26.7%)	4/22 (18.2%)	0.69
** Urgent CD**	11/15 (73.3%)	18/22 (81.8%)	0.69
**Post-partum hemorrhage **^**b**^	7 (8.8%)	11 (6.1%)	0.603

Data presented as mean ± SD, mean ± SD (median (Q1-Q3)) (for non-normally distributed parameters), n(%) or *n/N (%)*

*Note: IVF, in-vitro fertilization; CD, Cesarean delivery*

^**a**^ *Defined by 2 measurements of* > *38 °C or one measurement of* > *39 °C during labor*

^**b**^ *Defined as more than 500 ml in a vaginal delivery or more than 1000 ml in a cesarean delivery*

Mode of delivery also differed, with significantly lower rate of spontaneous vaginal delivery (58.8% vs. 72.5%, *p* = 0.03) and higher rate of operative vaginal delivery in the IVF group (22.5% vs.12.5%, *p* = 0.048) (Table 2).

Neonatal outcomes were comparable among the study groups; however, we observed a higher rate of umbilical artery pH < 7.1 in the IVF group (9.8% vs. 0.0%, *p* = 0.022) (Table 3).

**Table 3:** Neonatal outcomes of the study (IVF pregnancy) and control (spontaneous pregnancy) groups

Parameter	IVF pregnancy (*n*=80)	Spontaneous pregnancy (*n*=160)	*P* value
**No. of patients**	80	160	
**Birthweight (grams)**	2994.7±517.4	3123.1±500.0	0.065
**Low birth weight (<2500 gr)**	11 (13.4%)	15 (9.4%)	0.304
**Birthweight>90% **	2 (2.5%)	9 (5.6%)	0.345
**Shoulder dystocia**	1 (1.3%)	0 (0%)	0.333
**Apgar in minute 5**	9.3±1.4	9.7±1.2	0.013
**Apgar<7 in minute 5**	3 (3.8%)	4 (2.5%)	0.689
**Umbilical artery pH <7.1 at delivery**	4/41 (9.8%)	0/63 (0%)	0.022
**NICU admission**	4 (5.0%)	8 (5.0%)	1
**Antepartum/Intrapartum fetal death**	1 (1.3%)	1 (0.6%)	1

*Data presented as mean* ± *SD (median) or n (%) or n/N (%)*

*Note: NICU *neonatal intensive care unit

A logistic regression analysis for the dependent parameter of spontaneous vaginal delivery vs. operative delivery (instrumental vaginal delivery or cesarean delivery) was performed. The model was adjusted for the significant parameters detected in the univariate analysis: mode of conception – IVF, previous vaginal deliveries, induction of labor, prolonged second stage of labor and gestational week at delivery. This analysis revealed that spontaneous onset of labor (OR = 2.08; 95% CI = 1.07–4.05, *p* = 0.03) was positively associated with spontaneous vaginal delivery while prolonged second stage of labor (OR = 0.35; 95% CI = 0.13–0.95, *p* = 0.04), independently, was negatively associated with this outcome (Table 4).

**Table 4:** Multivariate analysis of parameters associated with spontaneous vaginal delivery in laboring young women

Parameter	OR (95% CI)	*P* value
**Previous vaginal deliveries**	6.45 (0.76-55.6)	0.088
**Conception by IVF**	0.83 (0.38-1.79)	0.626
**Gestational week**	0.83 (0.69-1.01)	0.06
**Spontaneous onset of labor**	2.08 (1.07-4.05)	0.03
**Prolonged second stage of labor **	0.35 (0.13-0.95)	0.039

*Note*: *IVF* In-Vitro Fertilization

## Discussion

In this study we present data on maternal and neonatal outcomes in young women who underwent IVF treatment. Our main findings include similar rates of antepartum complications (including preterm births, low birth weight, pregnancy related hypertensive disorders and gestational diabetes) with several differences in intra-partum parameters (prolonged second stage of labor, meconium-stained amniotic fluid, higher rate of operative vaginal delivery and decreased rate of spontaneous vaginal delivery). Newborns of young women also had increased prevalence of umbilical artery pH < 7.1.

Previous studies have reported increased adverse obstetric outcomes in young women. These include preterm delivery, low birth weight, eclampsia, intra-uterine fetal death, maternal anemia, postpartum depression, and maternal death [16–24]. In order to control for the effect of young age on obstetric outcomes we specifically chose a control group which had comparable age. This was done to isolate the effect which infertility and IVF treatment may have on obstetric outcomes in this age group.

A possible explanation for the comparable outcomes despite conception by IVF may stem from the highly selected population of young women who undergo IVF treatment. In general, this population, compared to very young women who conceive spontaneously, may have improved health insurance and access to healthcare facilities leading to increased compliance with antenatal care, resulting in improved outcomes.

Women in the IVF group did not have increased rate of ante-partum maternal complications. This contrasts with previous studies showing that women who conceived following IVF treatment are at increased risk of hypertensive disorders of pregnancy and gestational diabetes [3–7]. It appears that those complications are less common in the IVF-treated young population. Furthermore, there is an ongoing discussion regarding the source of such complications: the underlying condition causing infertility vs. the IVF treatment itself provided to women. In this study, majority of infertility diagnoses were attributed to male infertility. Hence, one can speculate that the comparable results in this population (gestational hypertension and gestational diabetes, among others) are due to lack of a female infertility condition related to compromised maternal health (such as ovulation disorders, polycystic ovary syndrome, etc.).

Our findings point towards the safety of IVF in young women with respect to ante-partum complications. While they should encourage clinicians to counsel women on the safety of IVF treatment in young age, these results must be reconfirmed on a larger scale. Creating a national registry of IVF treatments including obstetric outcomes could be an important step in obtaining high quality and reliable data. This in turn is likely to assist researchers in future studies pertaining to IVF treatment outcomes and their implications on labor and delivery.

As mentioned, women in the IVF group had an increased rate of operative vaginal delivery compared to the control group. This was at the expense of spontaneous vaginal delivery rates since cesarean delivery rates were similar between groups. Previous studies looking at obstetric outcomes following IVF have shown increased intervention rates such as induction of labor and cesarean delivery but not operative vaginal delivery [10]. This finding may be explained by a more aggressive approach implemented by physicians treating women for whom pregnancy was achieved by IVF.

The strengths of this study include the selection of a matched control group by age and parity, two major factors associated with obstetric and neonatal outcomes, especially in the young population. Moreover, the data presented in this study may assist in fertility counseling for very young women facing the option of IVF treatment.

This study has several limitations, among them is the relatively small sample size and retrospective construct. Few women were under the age of 20 making it difficult to reach conclusions regarding this age group. Additional limitations include the possible selection bias of young women who undergo IVF treatments and the lack of a subgroup analysis within the IVF study group regarding the parameters of infertility etiology and type of embryo transfer. These analyses were not performed due to small sample size of each subgroup within the IVF group (80 patients overall). Nevertheless, this is the first study to compare young women who underwent IVF to a similar aged control group.

In summary, young IVF patients have comparable pregnancy outcomes compared to their counterparts who conceive spontaneously. While recognizing that further studies focusing on this unique age group are needed, we hope these results will encourage clinicians to offer IVF treatment to young women in need of this intervention and aid in their counseling (Fig 1).

## Data Availability

The datasets used and/or analyzed during the current study are available from the corresponding author on reasonable request.
